# Preclinical evaluation of Insulin-like growth factor receptor 1 (IGF1R) and Insulin Receptor (IR) as a therapeutic targets in triple negative breast cancer

**DOI:** 10.1371/journal.pone.0282512

**Published:** 2023-03-15

**Authors:** Sandra Roche, Patricia Gaule, Deirdre Winrow, Nupur Mukherjee, Fiona O’Neill, Neil T. Conlon, Justine Meiller, Denis M. Collins, Alexandra Canonici, Mohammed Ibrahim Fawsi, Alejandra Estepa-Fernández, Stephen F. Madden, John Crown, Norma O’Donovan, Alex J. Eustace

**Affiliations:** 1 National Institute for Cellular Biotechnology, Dublin City University, Glasnevin, Dublin, Ireland; 2 Data Science Centre, Royal College of Surgeons in Ireland, Dublin, Ireland; 3 Department of Medical Oncology, St Vincent’s University Hospital, Dublin, Ireland; Florida International University, UNITED STATES

## Abstract

Triple Negative Breast Cancer (TNBC), a subtype of breast cancer, has fewer successful therapeutic therapies than other types of breast cancer. Insulin-like growth factor receptor 1 (IGF1R) and the Insulin receptor (IR) are associated with poor outcomes in TNBC. Targeting IGF1R has failed clinically. We aimed to test if inhibiting both IR/IGF1R was a rationale therapeutic approach to treat TNBC. We showed that despite IGF1R and IR being expressed in TNBC, their expression is not associated with a negative survival outcome. Furthermore, targeting both IR/IGF1R with inhibitors in multiple TNBC cell lines did not inhibit cell growth. Linsitinib, a small molecule inhibitor of both IGF1R and IR, did not block tumour formation and had no effect on tumour growth in vivo. Cumulatively these data suggest that while IGF1R and IR are expressed in TNBC, they are not good therapeutic targets. A potential reason for the limited anti-cancer impact when IR/IGF1R was targeted may be because multiple signalling pathways are altered in TNBC. Therefore, targeting individual signalling pathways may not be sufficient to inhibit cancer growth.

## 1. Introduction

Triple negative breast cancer (TNBC) is an aggressive disease with a high risk of recurrence, and a lack of recognised molecular targets for therapy [1]. Currently, there are a limited number of approved targeted therapies for the treatment of TNBC including PARP inhibitors (BRCA-mutated TNBCs) immunotherapy in combination with chemotherapy and the antibody drug conjugates sacituzumab govitecan and trastuzumab deruxtecan [2–4]. Therefore, there remains an urgent need to identify novel treatment options for these women. It has previously been demonstrated that women with high levels of insulin are at increased risk of developing breast cancer [5]. Early studies demonstrated that insulin receptor (IR) was both overexpressed and phosphorylated in breast cancers compared to normal tissue [3, 5]. The elevated phosphorylation and expression of IR were shown to correlate with increased age of the patient, increased tumour grade and to be associated with poorer overall survival [6, 7].

Insulin-like growth factor receptor 1 (IGF1R) and its signalling has been extensively studied in breast cancer and it has been implicated in resistance to hormone therapies, HER2 targeted therapies, and chemotherapy [8]. Previously, we have shown that IGF1R inhibition increases response to trastuzumab in HER2 positive breast cancer cells [9, 10]. Previous studies demonstrated that ligand bound estrogen receptor (ER) alpha is required for rapid activation of the IGF1R signalling cascade in ER positive breast cancer [11]. Further Sciacca et al., 1999, provided evidence for an autocrine IR/IGF-II signalling pathway in breast cancer cells, including TNBC cell lines, where the IR-A isoform binds IGF-II with an affinity close to that of insulin [12]. However, to date, clinical studies of IGF1R antagonism in ER positive breast cancer have been disappointing [13, 14]. Interestingly however, activated IGF1R has been associated with reduced disease free survival in TNBC [15], and has thus been assessed as a molecular therapeutic target in TNBC either alone [16] or in combination with other tyrosine kinase inhibitors; particularly those that target the PI3K/AKT pathway [17, 18].

Both the IR and the IGF1R belong to the same tyrosine kinase receptor subfamily and share structural homology in the tyrosine kinase domain [7]. Because of the inherent link between IR and IGF1R and the association with poor survival outcome in TNBC, several attempts have been made to target both IR and IGF1R to treat the disease. Firstly, Litzenburger et al., demonstrated that TNBC cell lines are sensitive to a dual IR/IGF1R small molecule inhibitor, BMS-754807 [16]. More recently linsitinib (OSI-906), a novel small-molecule dual IGF1R/IR kinase inhibitor, demonstrated anti-proliferative effects in vitro and in vivo in both stem like and colorectal cancer models [19]. Linsitinib has progressed to clinical evaluation in solid tumours, but, in unselected tumours, it has shown low clinical efficacy and toxicity [20–22]. Xentuzumab (BI 836845) is a fully humanised IgG1 ligand-neutralising antibody. Whilst it does not target IR or IGF1R directly, it binds both Insulin Like Growth Factor 1 (IGF-I) and Insulin Like Growth Factor 2 (IGF-II) thus preventing activation of the receptors. Xentuzumab has been shown to reduce both IGF1R phosphorylation and cellular proliferation in preclinical studies of lung cancer, Ewing’s sarcoma and multiple myeloma [23] and was well tolerated in a recent Phase I clinical study in solid tumours [24].

Our hypothesis was that inhibition of IR in addition to IGF1R may overcome poor efficacy issues related to previously tested IGF1R inhibitors. We aimed to evaluate whether inhibition of both IR and IGF1R was a rationale therapeutic approach to treat TNBC.

## 2. Materials and methods

### 2.1. Cell lines and reagents

MDA-MB-468, HCC1143, HCC1937, HCC70, CAL-851, HDQ-P1, HCC1806, CAL120, CAL51, BT549, Hs578T, MDA-MB-231, MDA-MB-157, HCC1187, BT20 cells were acquired from collaborators in UCLA. All cell lines were grown in RPMI-1640 media supplemented with 10% FCS and grown in a 37°C incubator maintained with 5% CO_2_. Linsitinib and NVP-BEZ-235 were obtained from Carbosynth whilst xentuzumab was obtained under Material Transfer Agreement from Boehringer Ingleheim. Cisplatin was obtained from St Vincent’s University Hospital, Dublin Ireland. All drugs, not already in clinical formulation, were reconstituted in DMSO and stocks of 10 mM were stored at -20°C. Cell lines used in these experiments were confirmed as mycoplasma free by regular testing. Cell lines were fingerprinted using STR profiling.

### 2.2. Gene expression data

BreastMark is a database based on an algorithm developed by Molecular Therapeutics for Cancer, Ireland (MTCI) [25]. Using the BreastMark database, comparative gene expression analysis of IR and IGF1R was performed by determining mRNA expression across different breast cancer subtypes using the ssp2006 classifier. The gene expression signatures were then compared with Disease Free Survival (DFS), Distant Disease Free Survival (DDFS) and Overall Survival (OS) of the breast cancer patients, which were part of the 26 datasets included in the analysis. Survival curves are based on Kaplan-Meier estimates and the log-rank P-value is shown for difference in survival. Cox regression analysis was used to calculate hazard ratios. The R package ‘survival’ was used to calculate and plot the Kaplan-Meier survival curve. All calculations were carried out in the R statistical environment [25].

### 2.3. Quantitative real time RT-PCR

The expression status of IR-A, IR-B, IGF1R and IGF-II mRNA was analysed in a panel of triple negative breast cancer cell lines (HDQP-1, MDA-MB-468, HCC1143, MDA-MB-231, BT20, CAL120, HCC70 and CAL51) and MCF10A which represented a non-cancer breast control. RNA was extracted using the mirVana™ miRNA Isolation Kit (Ambion, AM1560) according to the manufacturer’s instructions. Triplicate RNA samples were isolated from the cell lines at 70–80% confluency. cDNA was prepared from 2 μg of total RNA using a high-capacity RNA to cDNA kit (Applied Biosystems, Foster City, CA, USA), master mix solution and reverse transcription conditions described as per S2A and S2B Table. A non-target control (NTC) which was prepared without the RNA template, and a minus reverse transcriptase control (RTC) was prepared without the reverse transcriptase enzyme. Taqman® Real Time PCR analysis was performed for IR-A, IR-B, IGF1R and IGFII, with GADPH as an endogenous control, on a 7900HT fast real-time PCR instrument (Applied Biosystems). Real-Time PCR was performed by adding 19 μL of qPCR master mix to the relevant wells of a 96-well PCR plate. qPCR master mix consisted of 10 μL of Taqman Universal PCR Master Mix (Applied Biosystems), 8 μL of RNase-free water (Ambion) and 1 μL of the specific assay (primer set), per reaction. To this 1 μL of each cDNA sample was added. Biological triplicates of cDNA samples were analysed in triplicate for measurement of target gene expression and endogenous control (GAPDH). Relative expression was quantified by qRT-PCR relative to the human mammary epithelial cell-line MCF10A.

### 2.4. Proliferation assays

Proliferation was measured using an acid phosphatase assay. CAL51 were seeded at 1 x 10^3^, whilst Hs578T, HCC70, MDA-MB-468, HCC1143, HCC1937, CAL-851, BT549, MDA-MB-231, HCC1806, and BT20 were seed at 3 × 10^3^ cells/well, whilst CAL120, HCC1187, MDA-MB-157, and HDQ-P1 were seeded at 5 × 10^3^ cells/well in 96-well plates. Plates were incubated overnight at 37°C followed by addition of drugs at the appropriate concentrations (<10 μM) and incubated for a further 5 days until wells were 80% to 90% confluent. Two types of combination assays were used. Fixed combination, where a single concentration of each drug was combined and fixed ratio proliferation, where drugs are combined over a range of concentrations at a fixed relative ratio. Following five days of incubation with drug, the media was removed, and the wells were washed once with PBS. Paranitrophenol phosphate (PNP) (ThermoFischer) substrate (10 mM of PNP in sodium acetate buffer, pH 5.5) was added to each well and incubated at 37°C for 1–2 hours. 50 μL of 1 M NaOH was added and the absorbance was read at 405 nM (reference– 620 nM).

### 2.5. 3D growth assays

96 well plates were coated with 50 μL polyhema (Sigma Aldrich S3932 5 mg/mL w/v in 96% ethanol) and baked at 50°C for two days. Plates are stored at room temperature until required, for up to 6 months. Cells were seeded at a density of 3 x 10^3^ cells/well (except BT549 which were seeded at 1.5 x10^3^ cells/well) in 10% FCS with Matrigel™ (2% MDA-MB468 and BT549; 4% MDA-MB-231 and HDQ-P1) and incubated overnight at 37°C. Complete media (30 μL) with/without serial dilutions of drug, was added to the wells and incubated at 37°C for a further 5 days. 12 μL of PrestoBlue® Cell Viability reagent (10% of final volume) was added to the wells and incubated at 37°C for 2–8 hrs (MDA-MB-231 2 hrs; MDA-MB-468 4 hrs; BT549 6 hrs; HDQ-P1 8 hrs) and fluorescently measured at 535/590 nm excitation/emission wavelength on Biotek plate reader using KC4 software. A blank consisting of media and 2–4% Matrigel™ as appropriate was used to eliminate background. Percentage viability was calculated relative to untreated control. Each assay was performed in biological triplicate.

### 2.6. Protein extraction and Luminex Magpix® multiplex magnetic bead assays

Protein was extracted using RIPA buffer with 1× protease inhibitors, 2 mM PMSF and 1 mM sodium orthovanadate (Sigma-Aldrich) was added to cells and incubated on ice for 20 min. Following centrifugation at 10,000 rpm for 10 min at 4°C, the resulting lysate was stored at −80°C. Protein quantification was performed using the bicinchoninic acid (BCA) assay (Pierce).

Magnetic bead assays were performed on the Luminex® MagPix® System (Merck Millipore, 80–073) using Milliplex Map Phospho Mitogensis RTK Magnetic Bead 7-Plex kit (Merck Millipore, 48–672 Mag) and Milliplex Map phosphor Human SRc Family Kinase Magnetic Bead 8-Plex kit (Merck Millipore 48-650Mag).

Protein (1–10 μg) was diluted in appropriate volume of assay buffer (final volume: 25 μL/well) and the assay was performed as per the manufacturer’s instructions.

### 2.7. Implantation and treatment of HCC1143 xenografts in mice

Female SCID (CB17/lcr-Prkdc^scid^/lcrCrl) mice were obtained from Charles Rivers (UK), maintained in pathogen-free conditions and fed a standard diet. Mice were kept at 25°C, with a 12 hour light-dark schedule and free access to food and water. All experiments were approved by DCU Research Ethics Committee (DCUREC/2015/208) and the Health Products Regulatory Authority (HPRA) under project authorization AE19115/P009, were carried out in accordance with the relevant guidelines and regulations and in compliance with the ARRIVE guidelines.

HCC1143 cells were grown as cell line–derived xenografts models to test the efficacy of linsitinib on tumour formation and tumour growth. HCC1143 cells were grown as described above and were prepared for implantation in serum free RPMI 1640 media and injected sub-cutaneously on the left flank of the mice at 5 x10^6^ cells/mouse. Animals were randomised into three arms at the point of implant, with 12 animals each in the vehicle and linsitinib arms, and 9 animals in the tumour only (treatment naïve) arm.

Linsitinib (25 mg/kg) or vehicle (25 mM L-tartaric acid) were administered daily by oral gavage to determine the impact on tumour formation. Treatment commenced on day 4 following tumour induction and continued for 78 days. Treatment commenced prior to tumours becoming palpable.

Mice were monitored daily for signs of toxicity. Animal body weights were measured three times/week and tumour volumes were measured twice weekly, using calliper measurements. Tumour volume was calculated as ((L*H*D)/1.9). Maximum allowable tumour was no greater than 1600 mm^3^ nor any tumour dimension exceeding 15 mm. Tumour measurements were blinded to treatment group. Treatment regime was 5 days on with 2 days off. A one week drug holiday was given following three weeks on treatment. The mice were sacrificed by cervical dislocation after the last linsitinib treatment or at a point when a humane end point was reached, such as a loss of body weight exceeding 20% of weight at start of treatment.

Tumours were dissected on sacrifice and collected in 10% formalin or snap frozen in liquid nitrogen.

### 2.8. Statistical analysis

To assess the impact of IGF1R and IR expression on DFS, OS and DDFS, survival curves are based on Kaplan-Meier estimates and the log-rank P-value is shown for difference in survival. Cox regression analysis was used to calculate hazard ratios. The R package ’survival’ was used to calculate and plot the Kaplan-Meier survival curve. All calculations were carried out in the R statistical environment. IGF-II mRNA expression was compared between basal and non-basal TNBC cell lines using the Fisher’s exact test. The combination of xentuzumab and cisplatin or docetaxel in TNBC cells was compared using a Student’s T-test. Statistical significance for tumour growth and body weight changes was determined by two-way ANOVA with Tukey’s multiple comparison test using GraphPad Prism v8.

## 3. Results

### 3.1. Expression and clinical significance of IR and IGF1R in TNBC

**Fig 1:**
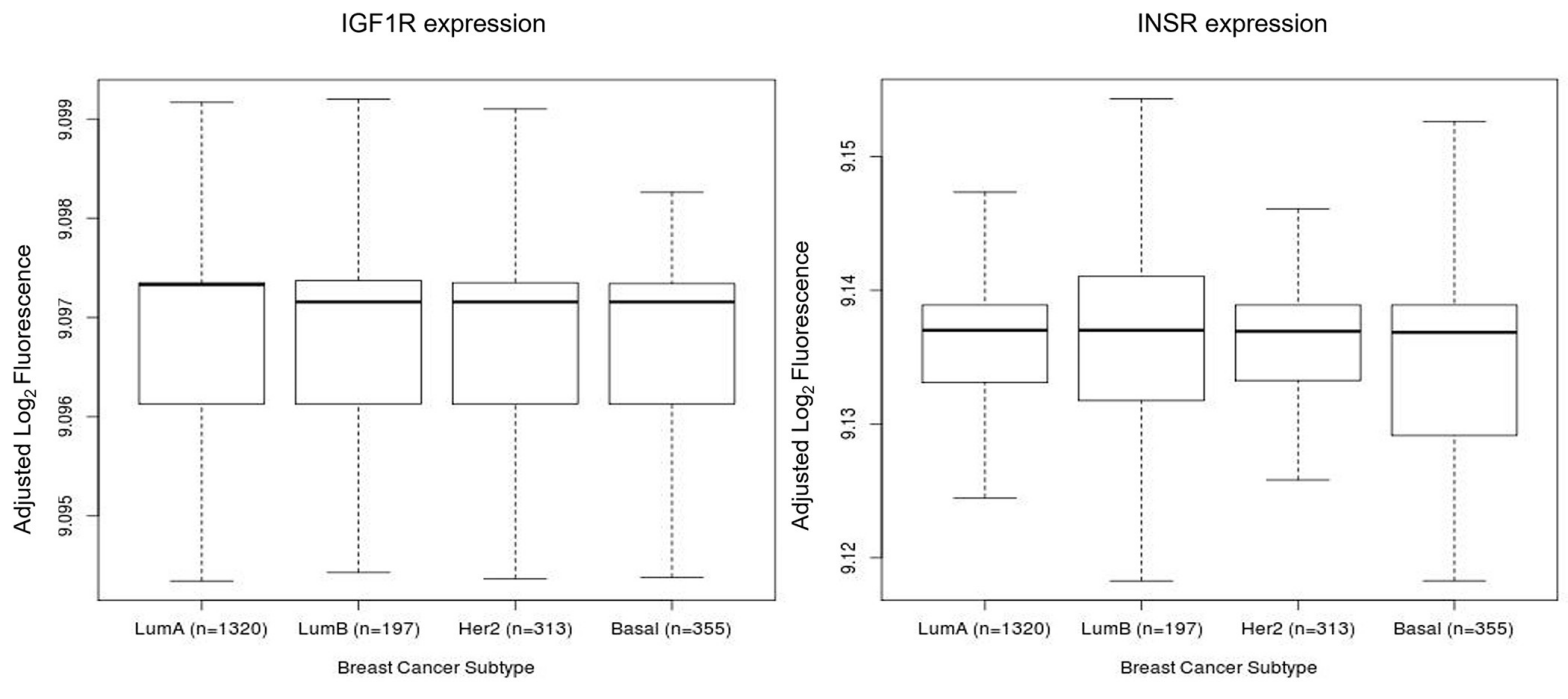
Expression of IGF1R and IR mRNA across the different breast cancer subtypes in the BreastMark database using the ssp2006 classifier.

**Fig 2:**
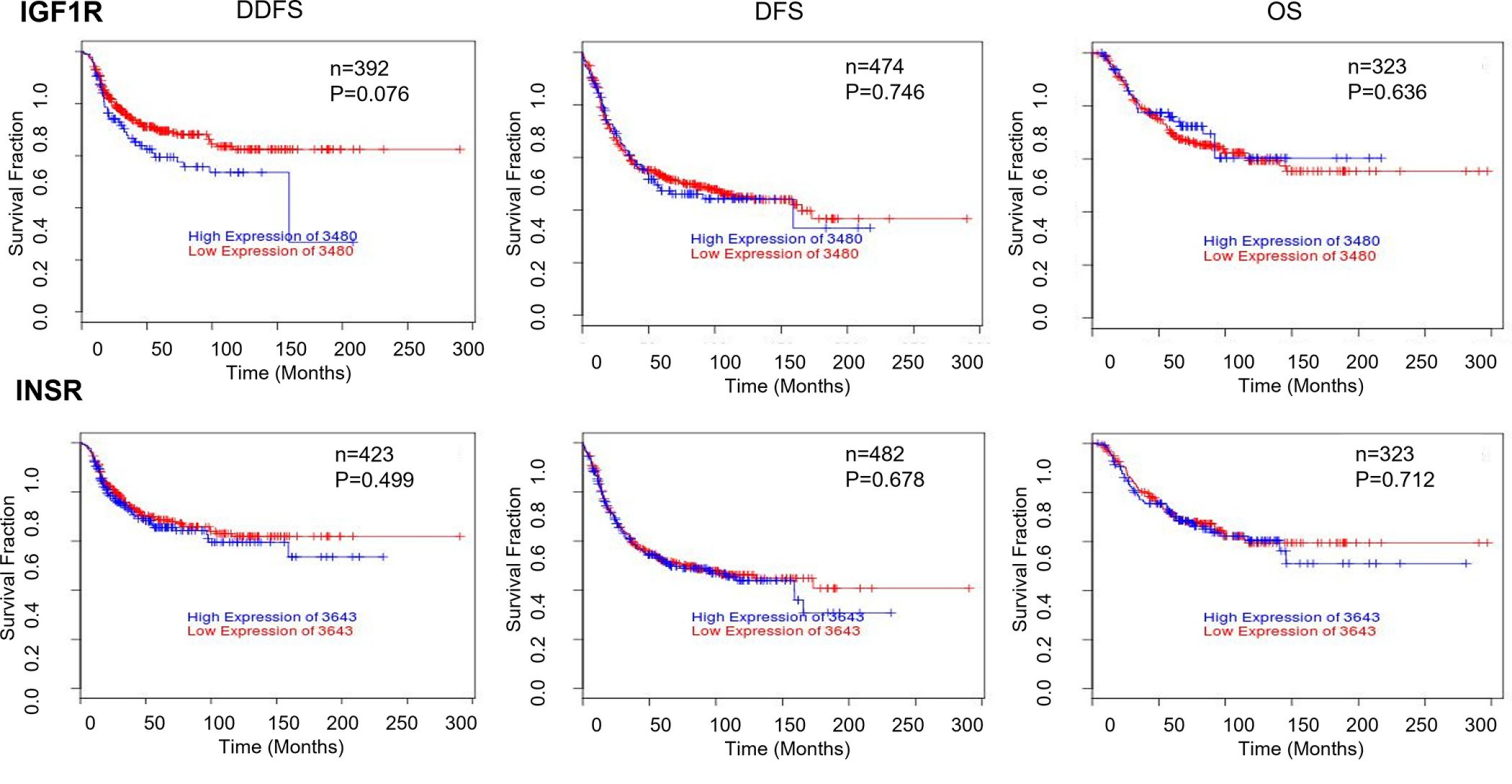
Kaplan Meier curves for distant disease free survival (DDFS), disease-free survival (DFS) and overall survival (OS) based on expression levels of IGF1R and IR mRNA in basal-like breast cancer, using the ssp2006 classifier on BreastMark.

Using the BreastMark platform and the ssp2006 classifier, we examined the differences in mRNA expression of IGF1R and IR in 2185 breast cancers, including 255 basal-like breast cancers, and found that neither expression of IGF1R nor IR was elevated in a specific subtype (Fig 1). Neither expression of IGF1R nor IR had any significant correlation with distant disease free survival (DDFS), disease free survival (DFS) or overall survival (OS) in a cohort of basal-like breast cancers (Fig 2). However, higher mRNA expression of IGF1R was associated with a worse distant disease free survival in all breast subtypes, although the result did not achieve statistical significance (p = 0.076).

### 3.2. In vitro impact of IGF1R and IR inhibition in a panel of TNBC cell lines

IR is alternatively spliced to form two isoforms A and B. IR (IR-A and IR-B) mRNA was detected in 10 of the 11 TNBC cell lines tested, whilst IGF1R mRNA was detected in the 11 TNBC cell lines and insulin like growth factor 2 (IGF-II) mRNA was detected in 6 of the 11 cell lines (Table 1). No significant differences were observed between the basal-like and non-basal-like cell lines for IR or IGF1R mRNA expression. However, interestingly, we found insulin-like growth factor 2 (IGF-II) mRNA was more frequently expressed in non-basal-like (80%, 4/5) compared to basal-like cell lines (20%, 1/5) (p = 0.08 Fisher’s Exact Test). We performed an Luminex Magpix® multiplex magnetic bead assays for IGF-I, IGF-II and IR in a TNBC cell line panel to determine their levels but found no correlation with response to any of the IGF1R/INSR inhibitors tested (S1 Table).

**Table 1:** IR-A, IR-B, IGF1R and IGF-II mRNA expression in a panel of triple negative breast cancer cell lines. Expression was quantified by qRT-PCR relative to the human mammary epithelial cell line MCF10A. Expression of either IR-A, IR-B, IGF1R or IGF-II gene determined relative to expression of the same gene in MCF10A cells. Relative quantity expressed as either + ≤1, ++ ≤10, +++ ≤100 or ++++ >100.

Cell line	Subtype	IR-A	IR-B	IGF1R	IGF-II
		mRNA Expression			
MDA-MB-468	Basal-like 1	++	+	++	
		(8)	(4)	10	0
HCC1143	Basal-like 1	+++	++++	++++	
		(14)	(105)	(905)	0
HCC38	Basal-like 1			+	
		0	0	(0.5)	0
HCC70	Basal-like 2	+	+	+++	
		(0.4)	(0.7)	(44)	0
HDQ-P1	Basal-like 2	++	+++	++++	++
		(5)	(22)	(101)	(6)
CAL120	Mesenchymal-like	++	+	++++	+++
		(9)	(2)	(143)	(11)
CAL51	Mesenchymal-like	+	+	++	++++
		(1)	(1)	(6)	(1850)
MDA-MB-231	Mesenchymal stem-like	+++	+++	++	
		(11)	(32)	(6)	0
MDA-MB-436	Mesenchymal stem-like	++	++	+++	+++
		(4)	(3)	(68)	(32)
HCC1187	Immunomodulatory	+++	++	+	+++
		(11)	(8)	(0.5)	(58)
BT20	Unclassified	+	+	+++	++++
		(0.45)	(0.8)	(78)	(1352)

To assess the impact of IGF1R inhibition on proliferation, we first tested the sensitivity of a panel of TNBC cell lines to the IGF ligand targeting antibody, xentuzumab, but it did not inhibit proliferation in the 8 cell lines assessed (Table 2). We also examined the tyrosine kinase inhibitor, linsitinib, which targets both the IGF1R and IR receptor in our panel of TNBC cell lines. Linsitinib inhibited growth in 4 of the 11 cell lines tested. An IC_50_ value was achieved in 3/7 basal like cell lines, whilst an IC_50_ value was only reached in 1/4 non-basal-like cell lines, at concentrations up to 10 μM (Table 2). In 3D-cultures, linsitinib showed similar efficacy to that observed in 2D-cultures in 3 out of 4 cell lines. However, in the HDQP-1 cells 3D growth inhibition was reduced compared to that observed in 2D-cultures (Table 2, S1 Fig).

**Table 2:** Sensitivity to xentuzumab and linsitinib in a panel of TNBC cells was assessed according to sections 2.4 and 2.5. A maximum of 10μM Linsitinib and 80μg/mL Xentuzumab was tested in serial dilution to obtain an IC50Average and standard deviations were calculated from triplicate independent assays. 3D assays were from at least duplicate assays. ‘-‘ indicates that no results have been obtained for that cell line under that test condition.

Cell line	Subtype	Xentuzumab	Linsitinib	Linsitinib	Linsitinib
		% inhibition @ 80 μg/mL	IC_50_ (μM)	% inhibition @ 10 μM	% inhibition @ 10 μM in 3D Growth
MDA-MB-468	Basal-like 1	7.1 ± 2.0	>10	6.8 ± 1.8	25
HCC1143	Basal-like 1	15.4 ± 1.0	6.6 ± 4.2	58.5 ± 5.7	-
HCC1937	Basal-Like 1	-	>10	14.9 ± 4.2	-
HCC70	Basal-like 2	4.2 ± 5.9	>10	22.6 ± 6.5	-
CAL-851	Basal-like 2	-	>10	12.1 ± 13.2	-
HDQ-P1	Basal-like 2	14.7 ± 3.2	2.8 ± 0.4	80.3 ± 3.7	35
HCC1806	Basal-like 2	-0.5 ± 2.9	9.5 ± 1.2	59.1 ± 7.4	-
CAL-51	Mesenchymal like	3.4 ± 6.6	>10	57.0 ± 10.2	-
BT549	Mesenchymal like	-	>10	8.0 ± 6.1	18
Hs578T	Mesenchymal stem like	-3.6 ± 3.3	>10	37.7 ± 2.4	-
MDA-MB-231	Mesenchymal stem like	5.8 ± 3.3	>10	21.5 ± 3.2	23

**Fig 3:**
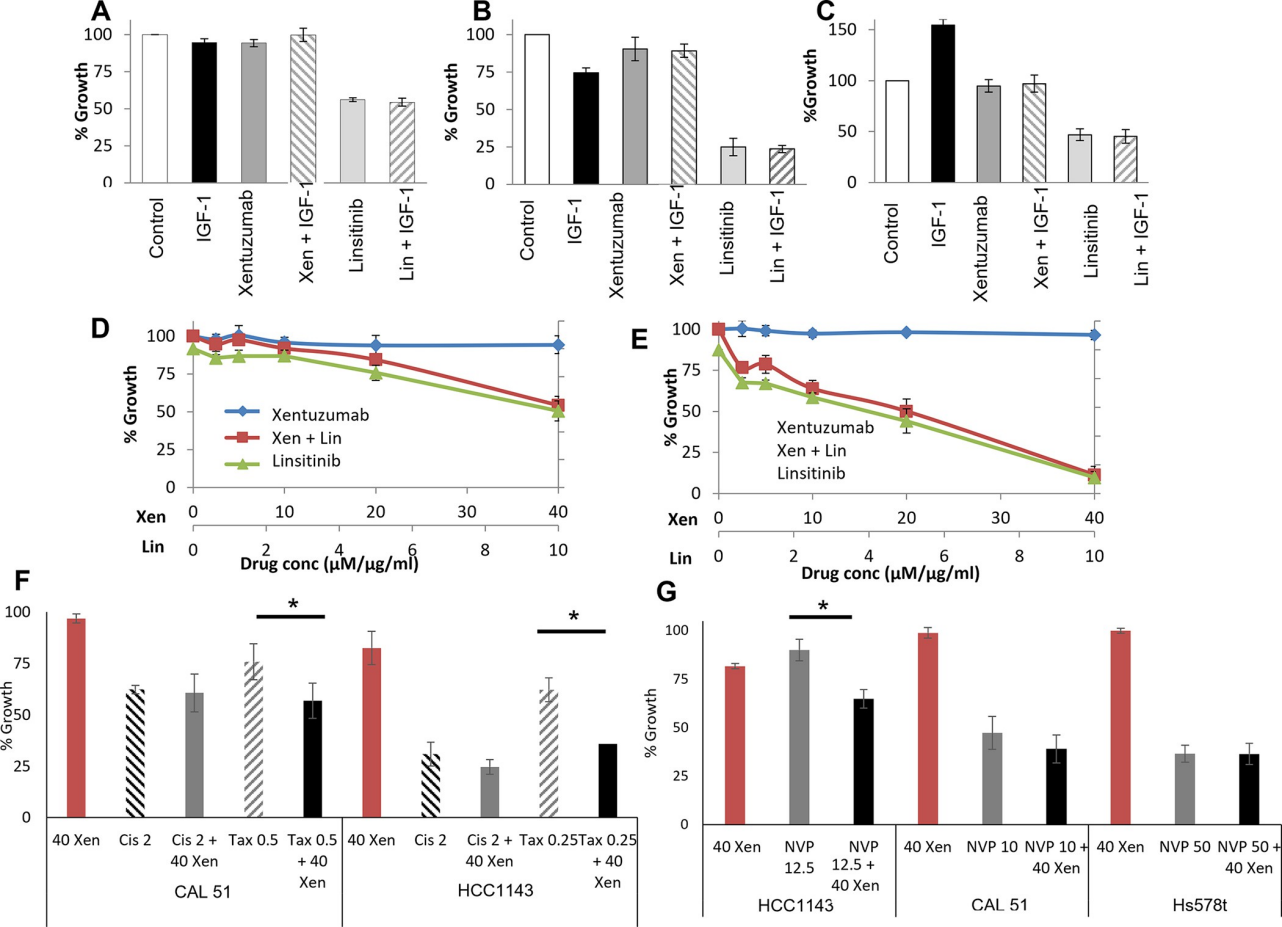
Proliferation assays in A) MDA-MB-231, B) HDQ-P1 and C) HCC1143. Cells were serum starved (2% FCS) for 24 hours followed by addition of IGF-I (50 ng/mL), with /without linsitinib (MDA-MB-231 10 μM; HDQ-P1; 3 μM; HCC1143 5 μM) or xentuzumab (40 μg/mL) (treated with 2% FCS). Proliferation assays in D) MDA-MB-231, E) MDA-MB-468 testing the combination of xentuzumab (BI-836845) and linsitinib in 2 TNBC cell lines. F) Proliferation assays in HCC1143 cells testing the anti-proliferative effects of combining xentuzumab (40 μg/mL) and cisplatin (2 μM) or docetaxel (0.5 nM) in the CAL-51 or HCC1143 cells. G) Proliferation assays in HCC1143, CAL-51 (PIK3CA Mut) and Hs578t (PTEN null) cell lines testing anti-proliferative effect of the combining xentuzumab and NVPBEZ-235 (PI3K/mTOR inhibitor). Standard deviations are calculated from triplicate independent experiments. ‘*’ indicates a p-value of <0.05 as calculated by Students T-test.

We also tested if ligand stimulation of TNBC cell lines which have high expression of IGF1R and/or IR, would stimulate growth and potentially sensitise the cells to inhibition by xentuzumab or linsitinib. In MDA-MB-231 and HDQ-P1 cells, IGF-I ligands did not stimulate proliferation of serum starved cells (Fig 3A and 3B). In HCC1143 cells, IGF-I ligand did stimulate growth by approximately 50%. In the ligand treated HCC1143 cells, xentuzumab and linsitinib blocked ligand stimulated growth (Fig 3C). We also tested the impact of IGF-II stimulation and found that it enhanced growth of HCC1143 cells. However, like IGF-I stimulation it did not enhance the anti-proliferative effects when combined with either linsitinib or xentuzumab (S2 Fig).

### 3.3. Combinatorial approach to inhibiting IGF signalling in vitro

To examine if linsitinib and xentuzumab could synergistically inhibit growth, two cell lines (MDA-MB-231, MDA-MB-468) were exposed to increasing concentrations of both agents alone and in combination (Fig 3D and 3E). However, in the MDA-MB-468 cell line (low expression of IGF1R or IR) and the MDA-MB-231 cell line (higher expression of IGF1R and IR), the combination of linsitinib and xentuzumab failed to improve the anti-proliferative effects, when compared to linsitinib alone.

We aimed to determine if xentuzumab could enhance response to treatment with either chemotherapy or PI3K inhibition in the CAL-51 and HCC1143 cell lines which are PIK3CA mutant. Xentuzumab was tested in combination with cisplatin, docetaxel and the PI3K inhibitor NVP-BEZ-235. The addition of xentuzumab to cisplatin did not significantly increase the anti-proliferative effect of cisplatin, however addition of xentuzumab to docetaxel significantly increased the anti-proliferative effects of docetaxel in both CAL-51 and HCC1143 cells (Fig 3F). Finally, when xentuzumab was combined with NVP-BEZ-235 in HCC1143 cells, increased growth inhibition (p = 0.003) was observed. HCC1143 cells are wild type for PIK3CA mutations and retain PTEN expression. The combination failed to improve growth inhibition in the CAL-51 (PIK3CA Mut) and Hs578t (PTEN null) cell lines (Fig 3G).

### 3.4. The effect of linsitinib on tumour formation and growth in vivo

The basal-like cell-line HCC1143 was selected for further in vivo analysis as the in vitro results showed linsitinib inhibited both ligand dependent and ligand independent growth in this cell line. HCC1143 cell-line derived xenografts were used to assess linsitinib growth inhibition in vivo. The rate of tumour formation was analysed by monitoring tumour detection rates. At 19 days post implantation 100% (9/9) untreated mice, 33.3% (4/12) of vehicle treated mice and 58.3% (7/12) of the linsitinib treated mice had palpable tumours. At 35 days after implantation 9/9 untreated mice, 9/12 vehicle treated mice and 11/12 linsitinib treated mice had palpable tumours (Table 3). Linsitinib treatment had no significant effect on tumour development when compared to the vehicle treated or the control group.

**Table 3:** Tumour take rate of HCC1143 xenografts as assessed for palpable tumours on day 19, 28, 33 and 35. Animals were randomised to each arm at the time of implantation and monitored for the detection of palpable tumours. Treatment commenced 4 days post implantation. Tumours became measurable at 33 days.

Days post implant	Vehicle	Linsitinib	Control
	n = 12	n = 12	n = 9
	% of mice with a palpable tumour		
19	33.3	58.3	100
28	58.3	83.3	100
33	66.7	91.7	100
35	75.0	91.7	100

**Fig 4:**
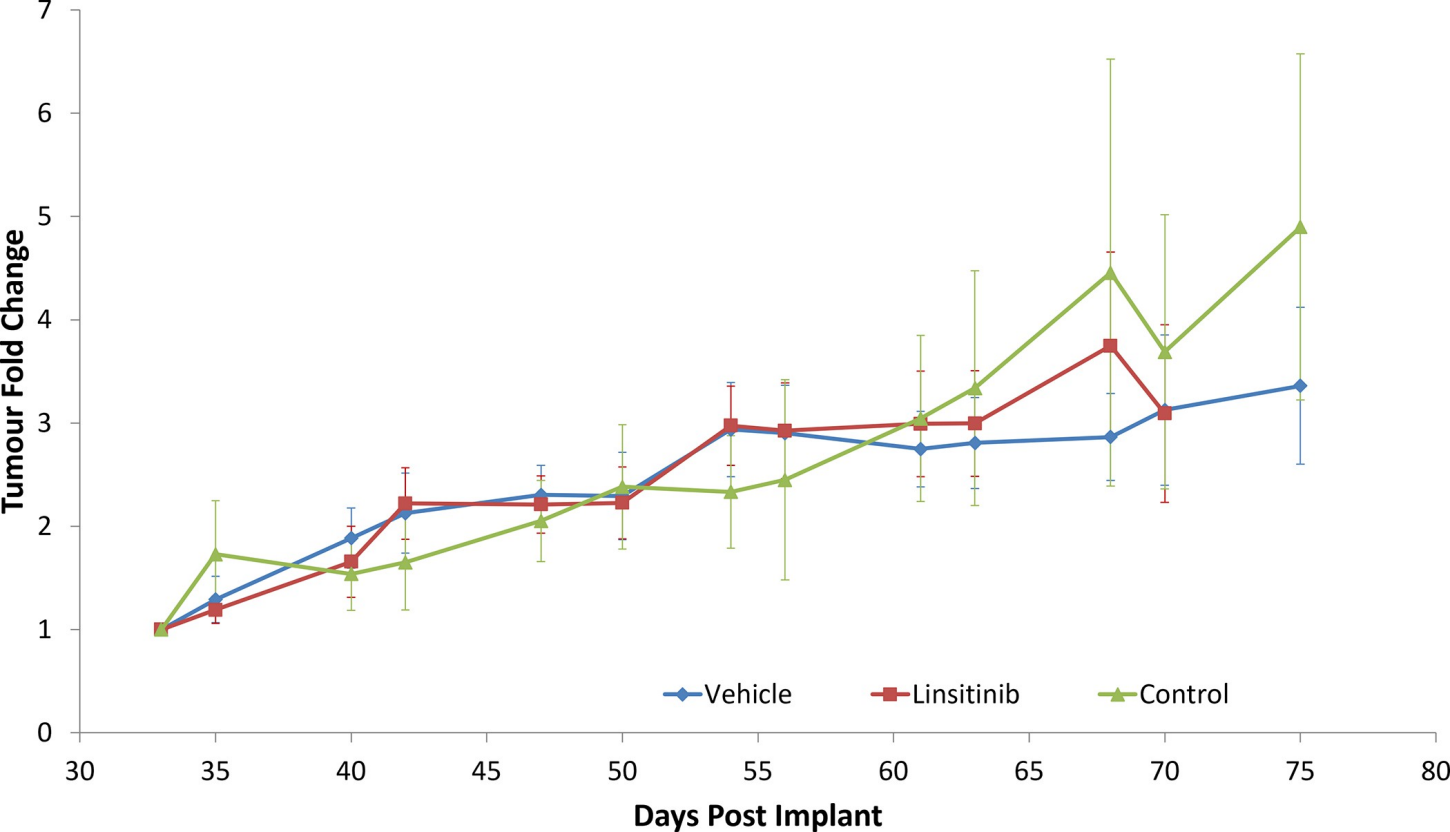
Effect of linsitinib and vehicle control on tumour volume, compared to untreated control. HCC1143 cells were implanted by subcutaneous injection into SCID mice. Animals were randomised to three arms; control, vehicle or linsitinib. Treatments, linsitinib (25 mg/kg) or vehicle were commenced 4 days post-implantation. Tumour volumes were assessed by callipers measurement. Tumour volumes were normalised to volume at day 33, as the starting point for tumour measurement, and represented as the mean and SEM. Tumour volumes arms were stopped when 5 animals remained on study.

Linsitinib and vehicle administration continued, and tumour measurement, by callipers, commenced on day 33. At day 35 the tumour volume averages were 30.8 +/- 14.8 mm^3^ (n = 9); 24.6 +/- 10.0 mm^3^ (n = 11) and 23.4 +/- 10.2 mm^3^ (n = 8) for the vehicle, linsitinib and control arms respectively. Tumour volumes were normalised to day 33 volumes and fold increase in tumour volume compared. No significant difference in tumour volume was detected between the treatment, control and vehicle arms (Fig 4). Linsitinib treatment at 25 mg/kg had no significant effect on tumour growth.

### 3.5. Systemic effect of linsitinib in vivo

**Fig 5:**
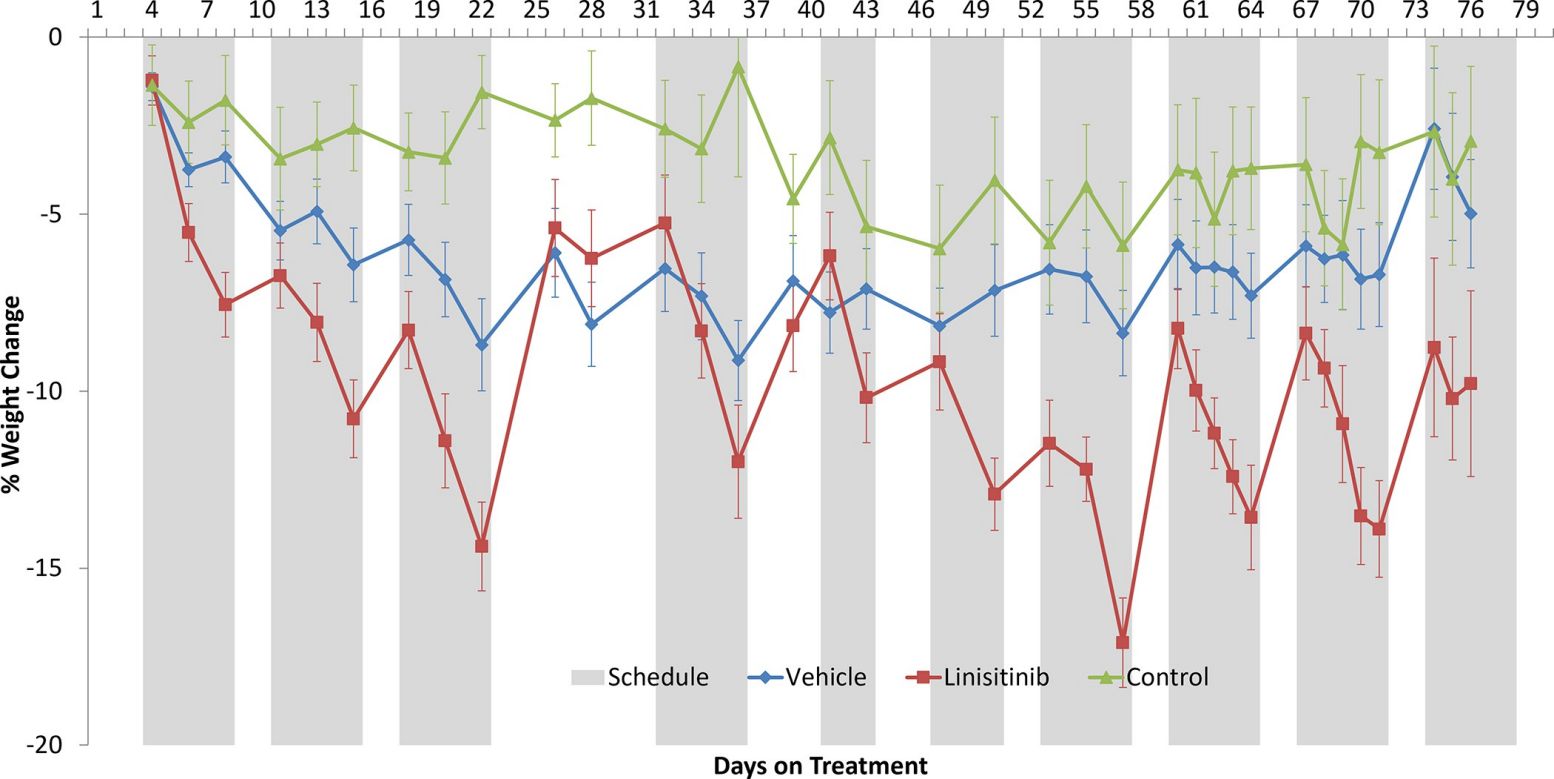
Effect of linsitinib, vehicle and untreated control on animal body weight on SCID mice implanted with HCC1143 cells. The treatment schedule is represented by the grey bars whereby white bars indicate days the animals did not receive treatment. Body weights were measured three times per week. Percentage weight change was monitored relative to the body weight at the time of implant. The mean percentage weight change and standard error of each group are shown. Statistically significant differences were calculated by two-way ANOVA using GraphPad Prism v8 and is represented by *or ** where * = <0.05, **<0.001.

As a marker for general health, the animals’ body weight was measured three times per week. Body weight change was calculated as a percentage change relative to each animal’s body weight at the time of implantation. Based on previous research by Mulvihill et al., a dose of 25 mg/kg was selected, as it was proven to reduce tumour growth but could avoid the most severe side effects associated with linsitinib treatment [19]. The administration of low dose, 25 mg/kg linsitinib, resulted in a significant and sustained weight loss. The therapy was stopped at day 22 and the animals were given a drug holiday with treatment re-commenced on day 32 until the end of study, with a consistent schedule of 5 days on, 2 days off. The percentage loss of body weight was statistically significant for the linsitinib treated group compared to the vehicle control on day 8 up to and including day 22 (Fig 5). At day 8, the linsitinib group showed a 6.7% drop in weight compared to 5.5% loss in the vehicle group (p = 0.0458). At day 22 the linsitinib group showed a 14.4% drop in body weight, compared to 8.7% in the vehicle control (p = 0.0035). Once treatment was stopped, the difference in the treated vs vehicle group was eliminated (day 26; linsitinib 5.4% weight loss, vehicle control 6.1% weight loss). This data shows that linsitinib has a significant impact on body weight in vivo. Cessation of linsitinib treatment resulted in recovery of body weight. This impact on body weight was not observed in the vehicle arm. While animals in the vehicle arm experienced minor weight loss compared to untreated control group, there was no statistical significance in the percentage body weight changes attributed to the vehicle.

## 4. Discussion

Both the expression and signalling activation of the IGF1R pathway have been extensively studied in breast cancer [8]. IGF1R signalling has been implicated in treatment resistance in breast cancer [10], whilst in TNBC, IGF1R has been associated with worse disease free survival [15]. IGF1R therefore has been the focus of numerous studies that aim to target IGF1R alone or in combination with other tyrosine kinase inhibitors; particularly those that target the PI3K/AKT pathway [17, 18].

In our study, whilst we found that both IGF1R and IR were detectable in all breast cancer subtypes, no subtype was associated with higher levels of IGF1R or IR expression. Furthermore, in a subtype analysis of basal-like breast cancer, which includes a majority of TNBCs, we found that neither IGF1R nor IR were associated with a worse outcome (overall survival, relapse free survival or distant disease free survival) using in-silico analysis. These findings raise questions regarding the oncogenic impact of IGF1R or IR expression in TNBC.

Several pre-clinical studies have indicated that IR signalling may play a role in resistance to IGF1R therapy. A study of 438 breast tumours found that IR was expressed in 49% of breast tumours compared to 32% for IGF1R [7]. IR is alternatively spliced to form two isoforms A and B, which we examined in our panel of TNBC cell lines. Previous studies of the IR mRNA levels of each isoform suggest that IR-A may be the more dominant isoform in breast cancer [26]. However, no significant differences were observed between the basal-like and non-basal-like cell lines for IR-A and B or IGF1R mRNA expression. A study published by Sciacca et al., provided evidence for an autocrine IR/IGF-II signalling pathway in breast cancer cells, including TNBC cell lines [12]. The 8 cell lines they tested expressed IR, predominantly the IR-A isoform, and 4/5 of the TNBC cell lines produced detectable levels of IGF-II. Unlike IR- B, the IR- A isoform binds IGF-II with an affinity close to that of insulin [27]. In our TNBC cell line panel, IGF-II mRNA was more frequently expressed in non-basal-like compared to basal-like cell lines.

To date, therapeutic strategies aimed at disrupting IGF signalling have largely focused on antibodies that target IGF1R. However, clinical studies of IGF1R antagonism in breast cancer have been disappointing. Further supporting the idea of dual targeting IGF1R/IR, the IR has been implicated in resistance to IGF1R monoclonal antibody therapy. Downregulation of IR by shRNA sensitised pancreatic and breast cancer cell lines, and pancreatic xenograft tumours, to IGF1R inhibition [28]. In the insulin-responsive 4T1 mouse model of breast cancer, the IGF1R/IR inhibitor BMS-536924 inhibited tumour growth, without significant hyperglycemia [29]. Litzenburger et al., examined the impact of targets IGF-1R and IR inhibitor, BMS-754807, in triple negative breast cancer models [16]. While, BMS-754807 targets IGF-1R and IR, it acts through mainly off target effects and phosphor-RTK array showed the activation of multiple protein kinases [30].

To test targeting of the IGF1R/IR axis, we tested the fully human monoclonal antibody xentuzumab which inhibits IGF signalling by selectively neutralising IGF ligands. Thus, it could be an effective and tolerable approach for inhibiting IGF-I and IGF-II signalling, without interfering with insulin/IR signalling. However, xentuzumab alone did not significantly inhibit proliferation in 6 of the 8 TNBC cell lines tested. Despite xentuzumab inhibiting IGF-I ligand stimulated proliferation in HCC1143 cells, xentuzumab failed to further inhibit proliferation relative to xentuzumab alone. Furthermore, in serum starved TNBC cells, stimulation with IGF ligands had no effect on proliferation rates in 2/3 cell lines tested. Only the HCC1143 cell line showed enhanced growth when treated with IGF ligands. These results possibly indicate a lack of dependence on IGF signalling in some TNBC cells. Identification of IGF-dependent tumours may be of relevance to xentuzumab efficacy in the clinic.

Linsitinib is a potent and highly selective tyrosine kinase inhibitor with similar biochemical potency against IGF1R and IR. We demonstrated that linsitinib inhibited growth of TNBC cell lines in both 2D and 3D assays, with potential preference for basal-like TNBCs, which correlates with work from Rigiracciolo et al., (2020) [31]. Consistent with the fact that HCC1143 cells responded to IGF1 stimulation, they also showed sensitivity to both xentuzumab and linsitinib. However, in 8 of the 11 TNBC cell lines tested the concentrations of linsitinib required to inhibit growth by 50% were greater than 10 μM.

To assess the impact of combined targeting of IGF ligands and the IGF/INS receptors, we tested the combination of linsitinib and xentuzumab in TNBC, but the combination failed to improve growth inhibition in vitro. Previous studies have demonstrated the benefit of combining xentuzumab with paclitaxel in xenograft and orthotopic implanted mouse studies. These studies used the 4T1 mouse model, which can also be used to study metastasis in vivo [32]. Our studies supported the use of this combination in TNBC, where combinations of xentuzumab and docetaxel enhanced growth inhibition in vitro, relative to either drug alone. However a Phase II clinical trial tested the combination of linsitinib and paclitaxel in epithelial ovarian cancer but found that the combination did not improve outcomes of patients relative to paclitaxel alone [33]. We also demonstrated that in a PIK3CA WT cell line, a combination of a PI3K inhibitor with xentuzumab enhanced growth inhibition compared to either drug alone. However, this effect was not observed in either a PIK3CA mutant cell line or a cell line with PTEN loss. In ER positive breast cancer, linsitinib abrogated increased phosphorylation of PI3K/MAPK proteins and Erα proteins as a result of the IGF-1R stimulation [34]. A recent Phase Ib/II of Xentuzumab plus everolimus and exemestane in hormone receptor-positive, locally advanced and/or metastatic breast cancer did not improve PFS in the overall population, leading to early discontinuation of the trial. However, the combination of xentuzumab/everolimus/exemestane in patients without visceral metastases showed evidence of PFS, leading to phase II XENERA™-1 trial (NCT03659136) [35].

To determine if greater anti-tumour activity would be observed in vivo, we tested the effect of targeting IR/IGF1R in a cell-line derived xenograft model of TNBC, using the HCC1143 cell line. In our study, linsitinib at 25 mg/kg did not prevent or delay tumour formation, but it did impact animal body weight. Additionally, continued dosing beyond tumour formation did not restrict tumour growth. Previously, linsitinib has been assessed in vivo to examine the effect on glucose uptake in non-small cell lung carcinoma xenografts, showing that at 60 mg/kg, linsitinib inhibited growth in IGF1R and IR positive tumours [36]. In IGF1R dependent colorectal cancer, linsitinib inhibited tumour growth in cell-line derived xenografts when treated with 40 mg/kg [37].

Linsitinib was investigated in a clinical trial with patients with advanced cancers, in combination with irinotecan; though no breast cancer patients were evaluated. Patients were treated with 400–450 mg linsitinib with irinotecan once daily on days 1–3, 8–10 and 15–17. Grade 1/2 weight loss was reported in 30% of patients [20]. In a Phase 2 study of men with metastatic castration resistant prostate cancer (mCRPC), receiving 150 mg orally twice daily on a 28-day cycle, 15 out of 17 enrolled patients discontinued treatment due to disease progression and adverse effects, demonstrating the lack of clinical efficacy targeting IGF1R [22]. In our study, mice exhibited significant weight loss while receiving linsitinib. The weight loss experienced by the mice is likely associated with drug induced hyperglycaemia (excess of glucose in the blood) [38]. Investigations in vivo showed that mice administered linsitinib (75 mg/kg) had higher levels of blood glucose; even a single administering of 7.5 mg/kg increased blood glucose [39]. In clinical studies, hyperglycaemia was frequently reported, with G3 hyperglycaemia reported by Macaulay et al., Jones et al., Carden et al., [40–42]. Tajima et al., showed systemic inhibition of phosphorylation of IR/IGF1R one hour after treatment with 45 mg/kg linsitinib, resulting in significant body weight loss [43]. Cumulatively these data suggest that the systemic inhibition of IR/IGF1R has a greater negative effect that any potential effect on tumour size or patient outcome.

## 5. Conclusions

To date the search for targeted treatments to improve the outcomes of TNBC patients have failed to provide benefit in the clinic. Both IR and IGF1R are associated with poor outcomes in TNBC, whilst IGF1R signalling has been implicated in resistance to both hormone and targeted therapy, as well as chemotherapy. In our study, we have shown that despite IGF1R and IR being expressed in all breast cancer subtypes including TNBC, in silico analysis demonstrated that neither IGFR1 nor IR expression are associated with outcome in this subtype. Furthermore, targeting the IR/IGF1R axis in TNBC failed to produce robust inhibition of cancer cell lines in vitro. Supporting the limited role of IGF1R and IR as a target in TNBC, linsitinib, an IGF1R/IR small molecule inhibitor, failed to block tumour formation in vivo and additionally, had no significant effect on tumour growth. The in vivo study utilised a cell line model that did respond to linsitinib and xentuzumab under ligand-stimulated conditions in vitro. Cumulatively these data suggest that while IGF1R and IR are expressed in TNBC, they do not represent good therapeutic targets. The issues may align with the fact that TNBC is a grouping of breast cancers that are negative for expression of specific receptors, unlike HER2 or ER positive tumours where a dominant signalling pathway is identified. TNBC is a grouping of breast cancers that are negative for expression of specific targetable receptors unlike HER2 or ER positive breast cancer, where a dominant pathway is identified. The cumulative results of our study suggest that while IGF1R and IR are expressed in TNBC, they do not represent a dominant signalling pathway and are therefore not good therapeutic targets. Bearing in mind that TNBC is a very heterogenous disease, with distinct molecular subtypes [44], future approaches to target IGF1R should focus on specific subtypes in which oncogenic dependence to IGF1R occurs rather than targeting all TNBCs.

## Supporting information

S1 Fig Sensitivity to linsitinib in a panel of TNBC cells as determined by the acid phosphatase assay, where standard deviations were calculated from triplicate independent assays. (DOCX)

S2 Fig Proliferation assays in HCC1143 after stimulation with IGF-I or IGF-II. Cells were serum starved (2% FCS) for 24 hours followed by addition of IGF-I or IGF-II (50 ng/mL), with /without linsitinib 5 μM or xentuzumab (40 μg/mL) (treated with 2% FCS). Standard errors represent the average results from triplicate independent results.
(DOCX)

S1 Table IGF-1, IGF-2 and INSR protein expression measured by LUMINEX multiplex bead array assays in a panel of triple negative breast cancer cell lines. Target quantity expressed as either + ≤100, ++ ≤200, +++ ≤500 or ++++ >500. ‘-‘not tested.
(DOCX)

S2 Table PCR conditions with (A) detailing the components of master mix solution for reverse transcription reaction and (B) detailing Thermo-cycler steps, temperature and duration for RT-PCR experiment.
(DOCX)

S1 Dataset (XLSX)
