# Supplementary material for: Preclinical evaluation of Insulin-like growth factor receptor 1 (IGF1R) and Insulin Receptor (IR) as a therapeutic targets in triple negative breast cancer
Source: PLoS One. 2023 Mar 15;18(3):e0282512. doi: 10.1371/journal.pone.0282512 (PMC10016661; doi:10.1371/journal.pone.0282512)
Supplement: S1 Fig — (DOCX) [file pone.0282512.s001.docx]

**Supplementary Figure 1:** Sensitivity to linsitinib in a panel of TNBC cells as determined by the acid phosphatase assay, where standard deviations were calculated from triplicate independent assays**.**
