# Supplementary material for: Preclinical evaluation of Insulin-like growth factor receptor 1 (IGF1R) and Insulin Receptor (IR) as a therapeutic targets in triple negative breast cancer
Source: PLoS One. 2023 Mar 15;18(3):e0282512. doi: 10.1371/journal.pone.0282512 (PMC10016661; doi:10.1371/journal.pone.0282512)
Supplement: S2 Fig — Cells were serum starved (2% FCS) for 24 hours followed by addition of IGF-I or IGF-II (50 ng/mL), with /without linsitinib 5 μM or xentuzumab (40 μg/mL) (treated with 2% FCS). Standard errors represent the average results from triplicate independent results. (DOCX) [file pone.0282512.s002.docx]

**Supplementary Figure 2**: Proliferation assays in HCC1143 after stimulation with IGF-I or IGF-II. Cells were serum starved (2% FCS) for 24 hours followed by addition of IGF-I or IGF-II (50 ng/mL), with /without linsitinib 5 μM or xentuzumab 40 μg/mL) (treated with 2% FCS). Standard errors represent the average results from triplicate independent results.
