# Supplementary material for: Preclinical evaluation of Insulin-like growth factor receptor 1 (IGF1R) and Insulin Receptor (IR) as a therapeutic targets in triple negative breast cancer
Source: PLoS One. 2023 Mar 15;18(3):e0282512. doi: 10.1371/journal.pone.0282512 (PMC10016661; doi:10.1371/journal.pone.0282512)
Supplement: S1 Table — Target quantity expressed as either + ≤100, ++ ≤200, +++ ≤500 or ++++ >500. ‘-‘not tested. (DOCX) [file pone.0282512.s003.docx]

**Supplementary Table 1:** IGF-1, IGF-2 and INSR protein expression measured by LUMINEX multiplex bead array assays in a panel of triple negative breast cancer cell lines. Target quantity expressed as either + ≤100, ++ ≤200, +++ ≤500 or ++++ >500. ‘-‘not tested.

| **Cell line** | **Subtype** | **Protein Expression** | | |
| --- | --- | --- | --- | --- |
|  |  | **IGF-I** | **IGF-II** | **INSR** |
| **MDA-MB-468** | Basal-like 1 | - | - | - |
| **HCC1143** | Basal-like 1 | ++++ | + | +++ |
| **HCC38** | Basal-like 1 | - | - | - |
| **HCC70** | Basal-like 2 | - | - | - |
| **HDQ-P1** | Basal-like 2 | ++ | ++ | +++ |
| **CAL120** | Mesenchymal-like | - | - | - |
| **CAL51** | Mesenchymal-like | - | - | - |
| **MDA-MB-231** | Mesenchymal stem-like | +++ | ++++ | ++ |
| **MDA-MB-436** | Mesenchymal stem-like | - | - | - |
| **HCC1187** | Immunomodulatory | + | ++++ | ++++ |
| **BT20** | Unclassified | - | - | - |
