# Supplementary material for: Preclinical evaluation of Insulin-like growth factor receptor 1 (IGF1R) and Insulin Receptor (IR) as a therapeutic targets in triple negative breast cancer
Source: PLoS One. 2023 Mar 15;18(3):e0282512. doi: 10.1371/journal.pone.0282512 (PMC10016661; doi:10.1371/journal.pone.0282512)
Supplement: S2 Table — PCR conditions with (A) detailing the components of master mix solution for reverse transcription reaction and (B) detailing Thermo-cycler steps, temperature and duration for RT-PCR experiment. (DOCX) [file pone.0282512.s004.docx]

**Supplementary Table 2A** Components of master mix solution for reverse transcription reaction

| **Component** | **Volume / Reaction**  **(µL)** |
| --- | --- |
| 10X RT buffer | 2.0 |
| 25X dNTP Mix (100 mM) | 0.8 |
| MultiScribe ^TM^ Reverse Transcriptase | 1.0 |
| RNase-free water | 4.2 |
| 10X RT random primers | 2.0 |
| **Total per reaction** | **10.0** |

**Supplementary Table 2B** Thermo-cycler steps, indicating the temperature and duration, for RT-PCR experiment

| **Step** | **Temperature**  **(°C)** | **Time**  **(minutes)** |
| --- | --- | --- |
| 1 | 25 | 10 |
| 2 | 37 | 120 |
| 3 | 85 | 5 |
| 4 | 4 | Hold |
